# Human Antigen R, a Myofibroblast-Specific Target in Treating Cardiac Fibrosis∗

**DOI:** 10.1016/j.jacbts.2024.05.001

**Published:** 2024-06-24

**Authors:** Abhay Srivastava, Sanjiv Dhingra

**Affiliations:** Regenerative Medicine Program, Institute of Cardiovascular Sciences, St. Boniface Hospital, Albrechtsen Research Centre, Department of Physiology and Pathophysiology, Rady Faculty of Health Sciences, Biomedical Engineering Program, University of Manitoba, Winnipeg, Manitoba, Canada

**Keywords:** cardiac fibrosis, heart failure, human antigen R, left ventricular remodeling, myofibroblasts

**Figure:**
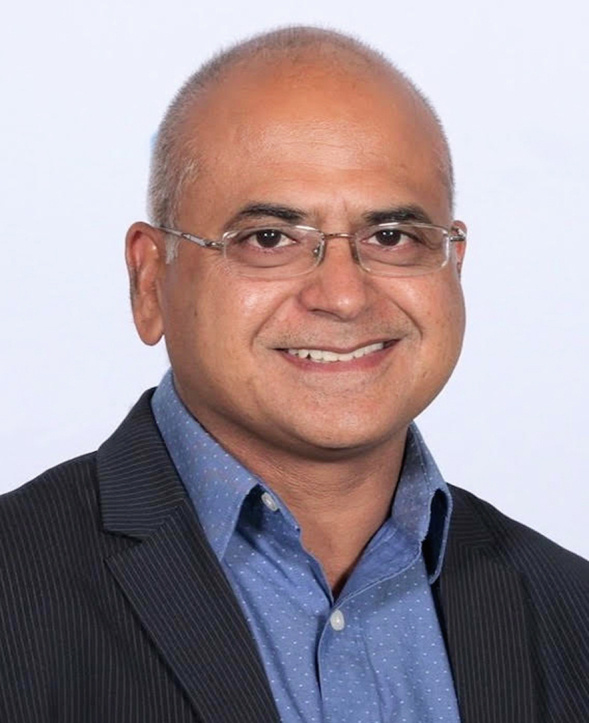

Fibrosis represents a significant cause of morbidity and mortality in cardiac patients. It is characterized by an irreversible alteration in myocardial tissue due to activation of fibroblasts into myofibroblasts and the accumulation of excessive extracellular matrix (ECM) proteins. Although replacing the dead cardiomyocytes by fibrotic tissue initially serves as a reparative mechanism by forming scar tissue and providing structural support to the heart, excessive collagen deposition ultimately hinders cardiomyocyte contractility and function, leading to systolic and diastolic dysfunction.^1^

Recently, RNA-binding proteins (RBPs) have garnered increasing attention for their role in cardiovascular diseases, primarily through their regulation of gene transcription by modulating messenger RNA (mRNA) biogenesis. Among these RBPs, human antigen R (HuR) has emerged as a key player in various cardiovascular pathologies, including myocardial ischemia, ischemia-reperfusion injury, atherosclerosis, and pulmonary arterial hypertension.^2^ HuR functions via binding to the 3′-untranslated region of target mRNAs, thereby promoting their stability and translation. Notably, HuR has been implicated in fibrosis regulation across multiple organ systems.

Recent studies have elucidated the role of HuR in activating cardiac fibroblasts into myofibroblasts, thus promoting cardiac fibrosis. Specifically, HuR has been shown to selectively bind to Wnt1-inducible signaling pathway protein-1 (Wisp1) mRNA in transforming growth factor β (TGF-β)–treated fibroblasts. Wisp1 expression was significantly up-regulated in fibroblasts isolated from ischemic areas of mouse hearts, indicating that HuR might be essential in TGF-β– activated cardiac fibroblasts via modulation of Wisp1 mRNA.^3^ However, despite this strong association of HuR and cardiac remodeling, the precise role of HuR in myofibroblast phenotype and function remains unclear.

In this issue of *JACC: Basic to Translational Science*, Patil et al^4^ investigated the role of HuR in cardiac myofibroblasts. Their findings revealed that inhibition of HuR in TGF-β–treated cardiac fibroblasts suppressed myofibroblast differentiation and proliferation. Further analysis showed that HuR stabilized cyclin D1 and cyclin A2 mRNA in activated fibroblasts, thereby promoting myofibroblast proliferation. These in vitro results suggest a direct positive correlation between HuR and cardiac fibrosis. This group has previously reported a predominant localization of HuR within fibroblast-like cells in the infarct region of ischemic hearts.^5^ To validate the direct role of HuR in myofibroblasts in vivo, Patil et al^4^ developed the first myofibroblast-specific tamoxifen-inducible HuR knockout mouse model. Using a transverse aortic constriction pressure overload injury model, they observed that deletion of HuR inhibited myocardial fibrosis by reducing ECM molecule expression and collagen deposition. Hence, this study conclusively establishes the pivotal role of HuR in myofibroblast-mediated cardiac remodeling and suggests that targeting HuR may offer therapeutic benefits in cardiac fibrosis and associated pathologies.

The predominant theme underlying pathologic fibrotic remodeling in heart injury is the activation of cardiac myofibroblasts. Therefore, for effective heart injury repair, it is crucial to explore strategies that can prevent activation of fibroblasts. Another critical consideration in treating cardiac fibrosis is determining the optimal therapeutic window, which takes into account factors such as myocardial structural integrity, ECM maturity, and excessive nondegradable ECM deposition. The study by Patil et al^4^ is a significant advancement in understanding the molecular translational regulation during cardiac fibrosis. It provides compelling evidence regarding the role of HuR in the activation of cardiac myofibroblasts. Through the creation of cardiac myofibroblast–specific HuR deletion models both in vitro and in vivo, the study offers focused insights into the molecular mechanisms involving HuR in regulation of cyclins D1 and A2. Moreover, this study presents tangible evidence that HuR deletion in myofibroblasts could mitigate cardiac fibrosis and improve heart function, thereby contributing to a better understanding of the optimal therapeutic window for treating cardiac fibrosis.

However, this study^4^ has its limitations and prompts further inquiry into the paradigm of cardiac fibrosis. Although classic targets for cardiac fibrosis consist of TGF-β, adrenergic receptor, and renin-angiotensin-aldosterone signaling,^6^ the current study^4^ primarily focuses on the mechanism of action of HuR and its downstream targets in response to TGF-β activation of myofibroblast proliferation and differentiation. However, whether HuR can mediate other signaling pathways in myofibroblasts remains unknown. In addition, HuR has been shown to stabilize the soluble guanylyl cyclase that produces nitric oxide, a vasodilator. Under hypoxic conditions, HuR expression in arterial smooth muscle cells is down-regulated, which in turn lowers soluble guanylyl cyclase stability and expression, thus causing vasoconstriction leading to pulmonary hypertension. HuR stabilizing activity in endothelial cells regulates the expression of CD62E and cathepsin S, which aids in degradation of elastin and collagen layers on the arterial walls, a vital process in regulating the pathogenesis of atherosclerosis. An increase in HuR expression in mouse cardiomyocytes has been observed during ischemia and reperfusion injury, where it has been shown to stabilize beclin mRNA, which leads to increased cell death.^7^ Therefore, the role of HuR in cardiomyocyte and endothelial cell function in the context of cardiac fibrosis requires further investigation. Also, inflammation and cardiac tissue injury are closely associated, and previous studies have reported that HuR can up-regulate the expression of proinflammatory cytokines, including tumor necrosis factor α,^8^ and increase the recruitment of macrophages in the injured heart.^9^ This is another potential area that necessitates exploration. Considering that many RBPs, including HuR, have been targeted using small molecules in cancer research,^10^ future development toward targeting RBPs using small molecule libraries could facilitate development of new therapeutic avenues for cardiac fibrosis as well.

In conclusion, the current study by Patil et al^4^ offers 2 notable contributions to the field of cardiac fibrosis. First, it identifies HuR as a therapeutic target for regulating myofibroblast phenotype and function. Second, the creation of a myofibroblast-specific HuR knockout mouse model itself provides a valuable tool for exploring HuR-mediated fibrotic responses in different heart injury models. These advancements hold promise for expanding our understanding of cardiac fibrosis and addressing numerous unanswered questions, thereby propelling the field forward.

## Funding Support and Author Disclosures

The authors have reported that they have no relationships relevant to the contents of this paper to disclose.
